# Therapeutic advances in muscular dystrophy

**DOI:** 10.1002/ana.23989

**Published:** 2013-10-09

**Authors:** Doris G Leung, Kathryn R Wagner

**Affiliations:** 1 Center for Genetic Muscle Disorders, Kennedy Krieger InstituteBaltimore, MD; 2 Departments of Neurology, Johns Hopkins School of MedicineBaltimore, MD; 3 Neuroscience, Johns Hopkins School of MedicineBaltimore, MD

## Abstract

The muscular dystrophies comprise a heterogeneous group of genetic disorders that produce progressive skeletal muscle weakness and wasting. There has been rapid growth and change in our understanding of these disorders in recent years, and advances in basic science are being translated into increasing numbers of clinical trials. This review will discuss therapeutic developments in 3 of the most common forms of muscular dystrophy: Duchenne muscular dystrophy, facioscapulohumeral muscular dystrophy, and myotonic dystrophy. Each of these disorders represents a different class of genetic disease (monogenic, epigenetic, and repeat expansion disorders), and the approach to therapy addresses the diverse and complex molecular mechanisms involved in these diseases. The large number of novel pharmacologic agents in development with good biologic rationale and strong proof of concept suggests there will be an improved quality of life for individuals with muscular dystrophy.

The muscular dystrophies are genetic disorders that cause progressive wasting and weakness of skeletal muscle. Although each of the individual muscular dystrophies is relatively rare, they collectively affect millions of people worldwide. The estimated prevalence rates of the most common forms of muscular dystrophy are 1 in 5,000 live male births for Duchenne muscular dystrophy (DMD) and between 1 in 7,000 and 1 in 20,000 for both facioscapulohumeral muscular dystrophy (FSHD) and myotonic muscular dystrophy.^1^–^5^ These disorders are often profoundly disabling due to loss of mobility and the increased incidence of medical comorbidities, such as heart disease and respiratory failure. The burden of disease and disability is carried not only by patients, but by caregivers and families. Although the cost of care for these disorders is likely to vary worldwide, the limited utilization data available make it clear that many in this patient population do not have access to the standards of care recommended for these diseases.^6^,^7^

Finding effective treatments for the muscular dystrophies has proven challenging, in part due to their heterogeneity. They are caused by mutations in >30 unique genes that produce myofiber degeneration and atrophy. Traditionally, muscular dystrophies have been defined by their clinical manifestations: distribution of weakness, mode of inheritance, onset and progression of disease, and distinctive nonskeletal muscle features. Genotyping of this patient population has challenged this convention, as it has been proven that the phenotypic spectrum of these disorders is much wider than previously suspected. Similar or identical mutations in a single gene can produce widely disparate phenotypes, even within the same family.^9^,^10^ Conversely, increasing numbers of distinct genes have been found to produce the same phenotype, such as the classic limb-girdle pattern of weakness. These discoveries have compelled the reclassification of genetic myopathies by their molecular deficits (eg, dystroglycanopathies, dysferlinopathies, laminopathies, ion channelopathies) in addition to their clinical features. This rethinking will ultimately improve our ability to develop targeted disease-modifying therapy and to define subsets of the population that are likely to respond to these treatments. It will also allow us to identify potential therapies for the muscular dystrophies through investigations of nonmuscle disorders that share the same genetic mechanism.

In this review, we will discuss therapeutic developments in 3 of the most common forms of muscular dystrophy. These disorders represent distinct classes of genetic disease: DMD is a single-gene, loss-of-function disorder, FSHD is an epigenetic disorder, and myotonic dystrophy is a repeat expansion disorder with toxic gain of function. By addressing the muscular dystrophies in this way, we hope to illustrate the need for mechanistic diversity in our research pursuits and in our approach toward treating the individual patient.

## Modern Understanding of Biology

### DMD

DMD and the milder, allelic Becker muscular dystrophy (BMD) represent a spectrum of disorders that stem from a loss of functional dystrophin protein. Since its discovery in the mid-1980s, there has been significant progress in determining the function of dystrophin and the biochemical consequences of its loss. Dystrophin not only has a structural role in linking the muscle cytoskeleton to the extracellular matrix, it also plays a prominent role in cell signaling and regulating muscle response to oxidative stress.^12^ The absence of dystrophin impedes the muscle's ability to tolerate conformational changes induced by contraction.^13^ The resultant muscle degeneration and inflammatory response produce a cellular environment in which adipocytes and fibroblasts proliferate and impair the regenerative capacity of muscle precursor cells.^14^ Advances in research in the dystrophinopathies have placed them at the forefront of exploration in the classic single-gene, loss-of-function disorders. Multiple therapeutic approaches to this disease (inducing dystrophin expression, preventing the downstream effects of muscle degeneration, and promoting muscle growth or replacement) are being explored in preclinical and clinical trials.

### FSHD

The past few years have proven to be a watershed period in the understanding of FSHD. Although a deletion in the D4Z4 macrosatellite repeat array of chromosome 4 was known to be associated with FSHD, we have only recently begun to understand how this change causes disease.^15^–^16^ It is now apparent that the underlying mechanism of FSHD involves a complex convergence of both genetic and epigenetic events. The contraction of the D4Z4 array is associated with hypomethylation of this region and chromatin relaxation that enables the transcription of *DUX4*, a gene that is normally suppressed after early development.^17^ *DUX4* mRNA is rapidly degraded unless it is transcribed in the context of specific haplotypes that contain a polyadenylation signal.^18^,^19^ Expression of full-length *DUX4* protein occurs in approximately 1 in 1,000 myocytes in vitro and induces transcription of germline genes and endogenous retrotransposons that are not normally expressed in adult skeletal muscle.^21^ The function of *DUX4* and how it contributes to muscle pathology are not fully understood. Full-length *DUX4* RNA is not always detectable in muscle tissue from FSHD patients, and it is present in some unaffected individuals with normal D4Z4 repeat arrays, suggesting that a second factor or mutation is required for pathogenesis.^21^–^22^ The recent discovery of a causative gene (*SMCHD1*) for FSHD2 (a disease identical to FSHD in terms of phenotype and chromosome 4q hypomethylation, but without contraction of the D4Z4 array) also supports a 2-hit hypothesis and further highlights the importance of both the genetic and epigenetic aspects of this disease.^23^,^24^

### Myotonic Dystrophy

The discovery of the microsatellite repeat expansion disorders provides further evidence of the importance of noncoding DNA and RNA function in muscle and other tissues. Myotonic dystrophy owes its multisystemic clinical phenotype to the widespread impact of repetitive code sequences on multiple cellular pathways. There have been 2 genetically distinct forms of myotonic dystrophy identified (type 1 is due to a trinucleotide repeat expansion of the *DMPK* gene on chromosome 19, and type 2 is associated with a 4-nucleotide repeat in the *ZNF9* gene on chromosome 3).^26^,^27^ These mutations interfere with RNA-binding proteins, leading to increased levels of CUGBP1/Elav-like family member 1 (CELF1) and decreased levels of muscleblind-like proteins (MBNLs), which are sequestered in ribonuclear foci. This results in mis-splicing of multiple genes, including *ClCN1*, which encodes the muscle chloride channel type 1 (leading to myotonia), the insulin receptor (leading to insulin resistance), and *TNNT2*, which encodes troponin T type 2 (potentially important in cardiac pathology),^29^–^32^ among others. In addition to inducing a “sliceopathy,” the repeat expansions may also induce disease through altered function of *DMPK* and *ZNF9*, dysregulated expression of neighboring genes, and repeat associated non-ATG translation.^33^–^36^ Recent studies have also begun to elucidate the role of repeat expansions in the central nervous system (CNS) manifestations of myotonic dystrophy, including cognitive dysfunction and sleep disorders.^37^,^38^

## Currently Available Therapies

Given the physical disability inherent in muscle disease and the multisystemic medical complications that can be associated with muscular dystrophies, the medical requirements in this patient population are formidable. The interdisciplinary management of physicians, therapists, counselors, and other specialists has extended the life expectancy of patients with muscular dystrophy considerably.^40^–^41^ Supportive care for the muscular dystrophy population may involve multiple nonpharmacologic interventions, including physical therapy, occupational therapy, orthopedic surgery, genetic counseling, invasive and noninvasive mechanical ventilation, and the use of implanted cardiac devices.^42^–^43^ However, pharmacologic disease-modifying treatments remain limited. In DMD, only corticosteroids have been shown to improve skeletal muscle strength and function in reproducible randomized controlled trials.^44^,^45^ Other pharmacologic therapies are primarily directed toward managing comorbidities (such as cardiomyopathy, osteoporosis, and respiratory failure).

## Therapeutic Pipeline in 2013

### DMD

#### Inducing Dystrophin Expression

Several therapeutic approaches to DMD are designed to promote endogenous dystrophin expression. One of the most novel and promising approaches is exon skipping, which utilizes antisense oligonucleotides (AONs) to promote alternative exon splicing and restore the open reading frame of dystrophin mRNA.^47^ The product of this technique is an altered but functional version of dystrophin that would predict a milder Becker, rather than Duchenne, phenotype. Two experimental drugs, eteplirsen (Sarepta Therapeutics, Cambridge, MA) and drisapersen (Prosensa Therapeutics, Leiden, the Netherlands and GlaxoSmithKline, Brentford, UK), are both designed to induce skipping of exon 51 (applicable to approximately 16% of DMD patients) and are being tested in phase II/III clinical trials.^48^,^49^ Although their oligonucleotide chemistries and side effect profiles differ, clinical trial data for both drugs show increased dystrophin expression in skeletal muscle biopsies of treated subjects. Even more impressively, subjects randomized to both study drugs had improved muscle function (measured by the 6-minute walk distance) compared to those randomized to placebo.^51^ A recent small molecule screen identified dantrolene (a drug approved by the US Food and Drug Administration for malignant hyperthermia) as a potential enhancer of exon skipping. Further studies in mice and reprogrammed human myotubes demonstrated that dantrolene given in conjunction with AONs increases levels of exon skipping by an order of magnitude and improves both dystrophin expression and muscle strength in *mdx* mice.^52^ Given the variability of dystrophin expression seen in exon skipping trials, the potential toxicities of AONs, and the high anticipated cost of AON administration, adjunctive measures that enhance exon skipping or lower the effective dose of these drugs will be clinically significant.

Approximately 10 to 15% of mutations in the DMD population are nonsense mutations that produce premature stop codons in dystrophin mRNA that result in no dystrophin expression or truncated proteins that are rapidly degraded. Aminoglycosides, such as gentamicin, allow the translational machinery to read through these stop codons, thereby producing full-length proteins.^53^–^54^ Although a 2-week trial of daily gentamicin in DMD/BMD failed to show dystrophin expression, a 6-month trial of weekly gentamicin demonstrated increased dystrophin expression in DMD boys with nonsense mutations.^55^–^56^ Due to dose-limiting toxicities of aminoglycosides, novel agents inducing readthrough have been sought. Ataluren (previously PTC124; PTC Therapeutics, South Plainfield, NJ) is an orally administered small molecule that is being evaluated for a number of disorders with nonsense mutations, including cystic fibrosis and DMD. Although ataluren has been described as increasing dystrophin expression and decreasing pathology in the *mdx* mouse, subsequent independent studies have questioned whether ataluren induces readthrough.^57^,^58^ Phase II clinical trials of ataluren in DMD have shown a good safety profile and a slower decline in 6-minute walk distances in a low-dose cohort (compared to placebo); however, no improvement in the 6-minute walk distance was observed in a high-dose cohort.^60^ A phase III trial of ataluren in ambulatory DMD patients is currently enrolling (ClinicalTrials.gov NCT01826487) and will hopefully provide definitive answers regarding its efficacy.

Introducing a functional dystrophin gene into dystrophic muscle cells has been a therapeutic goal since the discovery of the molecular basis of DMD in the 1980s. Numerous challenges have delayed the clinical implementation of this approach beyond initial expectations. However, using adeno-associated viral (AAV) vectors, investigators have succeeded in transducing both skeletal and cardiac muscle with modified forms of dystrophin (mini-dystrophins) in animal models of DMD.^61^–^62^ A phase I gene therapy trial using AAV 2.5 delivery of a mini-dystrophin gene supports the safety and tolerability of intramuscular delivery; however, dystrophin expression was limited.^63^,^64^ Since this first gene therapy trial in DMD, advances have been made in vector design, muscle-specific promoters, and mini-dystrophin constructs. It is anticipated that the safety of regional delivery will need to be established before systemic gene replacement in DMD can be achieved. The safety and feasibility of high-pressure transvenous limb perfusion with saline has recently been demonstrated in BMD, facilitating upcoming trials in the regional delivery of viral vectors.^66^

#### Muscle Regeneration and Replacement

Given the large number of individual mutations that can cause dystrophinopathy, it is important to explore therapeutic options that can improve muscle pathology but are not mutation-specific. Stem cell transplantation is an attractive option in this category that would be applicable not only to DMD but to the myriad other muscular dystrophies as well. Direct transplantation of myogenic precursor cells (MPCs) has proven difficult, and efforts to promote the migration, expansion, differentiation, and survival of these cells in vivo are ongoing.^67^ Transplantation of mesoangioblasts (vascular-derived stem cells) has demonstrated benefit in animal models of DMD and is currently in phase I human trials.^68^ Pharmacologic agents that induce muscle regeneration, such as myostatin inhibitors and insulinlike growth factor 1 (IGF-1), continue to be developed as solo therapy for the dystrophinopathies and other chronic myopathies. These agents will likely prove beneficial in conjunction with MPC transplantation not only for their ability to stimulate MPC proliferation and differentiation but also for their ability to reduce fibrosis.^69^,^70^

#### Modulating Signaling Pathways

Disruption of the dystrophin scaffold alters key signaling pathways necessary for muscle health and function. An especially important signaling protein is neuronal nitric oxide synthase (nNOS). One of the functions of sarcolemmal nNOSμ (the muscle-specific form of this enzyme) is local regulation of vascular flow during exercise; nitric oxide (NO) promotes smooth muscle relaxation and vasodilation. NO also regulates cardiac, skeletal, and smooth muscle contractile function and modulates the immune response in muscle. The major signaling pathway for NO is stimulation of cyclic guanosine monophosphate (cGMP) production by soluble guanylyl cyclase (sGC). In turn, cGMP directly activates protein kinase G (PKG) and certain ion channels. The NO-cGMP-PKG pathway is disrupted in muscular dystrophy, beginning with reduction in nNOS activity. Phosphodiesterases (PDEs) hydrolyze cGMP and turn down the gain of NO-cGMP signaling. Amplification of NO-cGMP signaling by administration of the PDE5A inhibitor sildenafil reduces functional deficits in cardiac performance and skeletal muscle pathology and function in aged *mdx* mice.^72^–^73^ The nNOS signaling pathway is a potential therapeutic target not only for DMD, but for multiple other muscular dystrophies in which nNOSμ is also mislocalized. Clinical trials are being conducted to investigate various phosphodiesterase inhibitors and novel agents that modulate the NO-cGMP-PKG pathway (ClinicalTrials.gov NCT01359670, NCT01168908, NCT01865084).^74^

#### Inhibition of Fibrosis

One of the late effects of muscle degeneration in DMD is replacement of muscle with fat and fibrosis.^75^ Fibrosis impedes muscle regeneration and may make several of the above therapeutics ineffective. Mechanisms to prevent or possibly reverse fibrosis include myostatin inhibition, transforming growth factor β (TGF-β) inhibition, and halofuginone, a novel drug that may exert its effects through the TGF-β pathway.^76^–^77^ Several clinical trials are planned or underway to determine the efficacy of compounds that are directed at inhibiting the formation of fibrotic tissue (ClinicalTrials.gov NCT01847573).

### FSHD

The identification of the *DUX4* gene as a mediator of disease in FSHD opens up new possibilities for targeted therapy. *DUX4* encodes a transcription factor that is believed to be involved in myogenic differentiation, and its anomalous expression in adult muscle in FSHD provides an attractive target for knockdown or inhibitory therapies. The role of DNA methylation and chromatin restructuring in FSHD also introduces the possibility that therapies that are being investigated in other epigenetic disorders will have utility in the treatment of FSHD.^78^ Clinical trials are being considered to determine the efficacy of several pharmacologic compounds—including selective androgen receptor modulators, myostatin inhibitors, and troponin activators—in the FSHD population. Although these therapies are not targeted toward the genetic mechanism that produces FSHD, there is evidence that they may promote muscle growth and/or improve skeletal muscle function.

### Myotonic Dystrophy

The pathology induced by DNA and RNA expansion in the myotonic dystrophies may be uniquely suited to therapeutic modification through antisense technologies. Some ways in which antisense therapy could reverse the pathology of myotonic dystrophy include: neutralization of binding sites for RNA-binding proteins, promoting targeted degradation of repeat expansion mRNA, and reduction in the size of the trinucleotide expansion. The latter mechanism is of particular interest, as the number of CTG repeats in the *DMPK* gene correlates with some aspects of disease severity in type I myotonic dystrophy.^79^ Therapies may also be directed toward the known downstream regulators of disease, such as *MBNL1* and *CELF1*.^80^ In considering potential therapies for myotonic dystrophy, however, it is important to recognize that the CNS manifestations of this disease (executive dysfunction, intellectual disability, sleep dysregulation), are frequently more disabling than muscle weakness.^81^–^82^ In this sense, investigation in the treatment of the myotonic dystrophies may benefit from growing research in other genetic disorders that affect the CNS (spinal muscular atrophy, amyotrophic lateral sclerosis/frontotemporal dementia secondary *C9orf72* mutations, Huntington disease, and the spinocerebellar ataxias).^83^–^84^

## Unmet Needs

As with all diseases that are severely debilitating and incurable, the need for treatment options in the muscular dystrophy population is manifest. As new therapeutic options enter into development, both investigators and patient groups recognize the need to facilitate and expedite clinical trials. To further this goal, it will be important to characterize the current natural history of these diseases and to develop outcome measures specific to the muscular dystrophy population. The most appropriate outcome measures will likely include clinical measurements and patient-reported outcomes as well as more novel imaging and biochemical disease markers. The identification, organization, and stratification of patient groups through registries and the collection of clinical and genotype data will also be important aspects of clinical trial preparedness.^85^–^86^ Further research on medical management and standards of care is also a significant unmet need in the muscular dystrophy population. There is considerable variability in the clinical management of these diseases, and studies are underway to help us better understand the risks and benefits of medical interventions (such as the various corticosteroid dosing regimens) and to make better-informed treatment recommendations.^87^

The management of the muscular dystrophies relies heavily on nonpharmacologic interventions that span multiple disciplines. Of particular interest to this patient population is the role of physical therapy, exercise, and other muscle stimulation techniques that can utilize endogenous mechanisms of muscle growth.^88^–^89^ This issue is controversial, as efforts to increase muscle strength through exercise may be countered by the risk of overuse and excessive breakdown of muscle that has limited regenerative capacity.^90^–^91^ Numerous studies have examined a variety of exercise programs in small numbers of patients with various types of muscular dystrophy, and there is evidence that some of these programs can be performed safely and at least temporarily improve muscle function.^92^–^93^ However, the lack of uniformity in exercise protocols and outcome measures has been a limiting factor in the development of widely applicable exercise guidelines.^94^–^95^ Although there is a demand for guidance on the relative benefits and potential harms (both medical and financial) of the numerous physical therapy options that confront patients, many of these interventions cannot be easily studied and measured under the methodologies that produce the highest levels of evidence (adequately powered, randomized placebo-controlled trials). Greater ingenuity in study design and data analysis is needed to understand their contribution to muscle health and to formulate guidelines on their use.

## Possible New Directions for Research

It is important to recognize that the optimal treatment for patients with muscular dystrophy may not take the form of a single drug or treatment modality, but rather a combination of treatments that address different pathological processes or act synergistically within the same pathway. Some of these possible complementary approaches are discussed here.

An important finding from a recent gene therapy trial (using AAV vectors carrying mini-dystrophin) was that an immune reaction to dystrophin occurred in some patients.^64^,^96^ Two of 6 subjects had dystrophin-specific T-cell responses prior to treatment consistent with priming, perhaps by rare revertant myofibers expressing dystrophin. As yet, there have been no reports of this phenomenon in other DMD trials (including those for exon skipping). However, if dystrophin-specific T-cell responses are found to occur frequently with induced dystrophin expression, it has broad implications not only for gene therapy, but for other genetic modifications and stem cell transplantation. An immune response to the vector capsid or transgene need not be insurmountable given the many tolerable immunomodulatory and immunosuppressive treatments available.

Increasing evidence in stem cell research has suggested that numerous factors in the local milieu promote or impair successful transplantation. Modulating the immune response against MPC grafts while providing a hospitable environment will improve the survival, migration, and differentiation of transplanted cells.^98^–^99^ Factors that inhibit fibrosis and promote muscle growth (such as myostatin inhibitors and IGF-1) may complement and enhance the efficacy of MPC transplants. The use of 3-dimensional scaffolds may also allow MPCs to differentiate into myotubes more successfully than single cells dispersed within fibrotic muscle tissue. Bioengineering of muscle tissue is advancing at an exciting pace as a treatment for traumatic injuries and has the potential to benefit those with muscular dystrophy as well.

In addition to continued pursuit of safe and effective pharmacologic agents that induce readthrough of premature stop codons, the field needs a better understanding of nonsense-mediated decay as it relates to the dystrophin transcript. Nonsense-mediated decay is a system of RNA surveillance that causes the degradation of mRNA with premature stop codons, abnormally spliced mRNA, and selected full-length mRNA transcripts. This is a highly conserved regulatory function designed to prevent expression of aberrant proteins and mediate the cellular response to stress.^100^ The rapidity with which nonsense-mediated decay occurs may limit the availability of transcripts that are targeted by drugs that promote nonsense readthrough. If so, inhibition of nonsense-mediated decay may be needed for these therapies to be efficacious. Although compounds that inhibit nonsense-mediated decay in vitro have been identified, they have not yet been tested for this purpose in humans.^101^

## Conclusions

The future of muscular dystrophy research promises to be both dynamic and productive, as great strides have been made in our understanding of the mechanisms underlying these diseases. These advances in knowledge are reflected in the proliferation of clinical trials and observational studies directed toward clinical trial preparedness. Muscular dystrophy patients now have an unprecedented number of pharmacotherapeutic trials available to them. In a field where the traditional approach to treatment has often been one of resignation and sympathetic fatalism, this new opportunity for optimism and intervention is both revolutionary and welcome.

## Potential Conflicts of Interest

Nothing to report.
